# Clinical prognostic value of OSGIN2 in gastric cancer and its proliferative effect in vitro

**DOI:** 10.1038/s41598-023-32934-5

**Published:** 2023-04-08

**Authors:** Peipei Wang, Ying Zhu, Xinru Jia, Xiangchang Ying, Leitao Sun, Shanming Ruan

**Affiliations:** 1 Department of Medical Oncology, The First Affiliated Hospital of Zhejiang Chinese Medical University (Zhejiang Provincial Hospital of Chinese Medicine), Hangzhou, 310006 China; 2 Zhejiang Key Lab of Prevention, Diagnosis and Therapy of Upper Gastrointestinal Cancer, Zhejiang Cancer Hospital, Hangzhou, 310022 China; 3 The First School of Clinical Medicine, Zhejiang Chinese Medical University, Hangzhou, 310053 China; 4 Academy of Chinese Medical Science, Zhejiang Chinese Medical University, Hangzhou, 310053 China

**Keywords:** Gastrointestinal cancer, Tumour biomarkers, Biomarkers

## Abstract

This study explored the promoting effect of oxidative stress-induced growth inhibitor family member 2(OSGIN2) on gastric cancer (GC) through public databases and in vitro experiments. The potential relationship between OSGIN2 expression, prognosis, functional enrichment of associated differential genes, immune infiltration, and mutational information in gastric cancer were comprehensively investigated using bioinformatics analysis. OSGIN2 was knocked down using small interfering RNA (siRNA) transfection for subsequent cell function testing. The results showed that gastric carcinoma cells and tissues contained high levels of OSGIN2, which was associated with a poor prognosis for GC patients. It was important in the cell cycle, autophagy, etc., and was related to a variety of tumor-related signal pathways. Knockdown of OSGIN2 inhibited tumor cell proliferation and contributed to cell cycle arrest. It was also correlated with tumor immune infiltrating cells (TILs), affecting antitumor immune function. Our analysis highlights that OSING2, as a new biomarker, has diagnostic and prognostic value in gastric cancer and is a potentially effective target in GC treatment.

As a malignant tumor, gastric cancer (GC) originates in the epithelial cells of the gastric mucosa. Despite the fact that its incidence and mortality rates have decreased globally over the past 50 years^1–3^, GC remains the world’s fifth most common type of cancer and the fourth major cause of cancer-related death. On the basis of the 2020 Global Cancer Statistics, there were more than a million cases of gastric cancer newly diagnosed and 760,000 deaths in 2020^4^. It was predicted that there would be 26,380 newly diagnosed cases and 11,090 deaths across the United States in 2022^5^. Helicobacter pylori infection is considered to be a major factor in GC^6^. In addition, drinking, smoking, and eating preserved foods are all known risk factors for GC^7^. At present, the main treatment strategies for GC are surgical resection, adjuvant chemotherapy, etc.^8^, but these are not ideal for the treatment of advanced tumor invasion and metastasis. For GC, the five-year survival rate is under 30^9,10^. Consequently, it is necessary to find prospective biomarkers and important targets that can predict GC malignant progression, as well as to develop safe and effective drugs to inhibit tumor invasion and metastasis.

Oxidative stress-induced growth inhibitor family member 2(OSGIN2), also named C8orf1/HT41, regions on chromosome 8q21.3 and adjacent to the gene for Nijmegen breakage syndrome. It encodes a 56.7 kDa protein with 505 amino acids^11,12^. Currently, there are few reports on the molecular mechanism of OSGIN2, and its potential role in GC treatment has not been explored. However, there is speculation that OSGIN2 may be involved in cancer development. Breast cancer cell lines were discovered to express OSGIN2 at a higher level than normal tissue cells, with high levels of DNA amplification and homozygous deletions^13^. Defamie V et al.^14^ observed upregulated OSGIN2 expression in liver biopsies with poor initial graft performance and speculated that OSGIN2 may be engaged in the process of cell meiosis or germ cell maturation. PGC-1-associated cofactor, a protein maintains mitochondrial homeostasis and links the mitochondrial state to the cell cycle, is associated with the occurrence and progression of cancer^15^ and is also related to prognosis^16^. Overexpressed OSGIN2 was detected when the expression level of the PGC-1-associated cofactors was suppressed^17^, which was related to gastric cancer^18^. However, nothing is known about how OSGIN2 works biologically in GC, which remains to be explored.

To this end, we compared OSGIN2 expression in gastric cancer and normal tissues using the TCGA and HPA datasets, and analyzed the clinical relevance between differences in OSGIN2 expression and prognosis in gastric cancer by the Kaplan–Meier plot database. Then, we validated the results on a variety of human gastric cancer cell lines by RT-qPCR. The pro-proliferative role of OSGIN2 was clarified by CCK8, clone formation, EdU, and other experiments. We further explored more possible functions of OSGIN2 in GC by KEGG, GSEA, etc., and looked for its effects on immune cell infiltration and gene mutations. The design is shown in Fig. 1.

**Figure 1:**
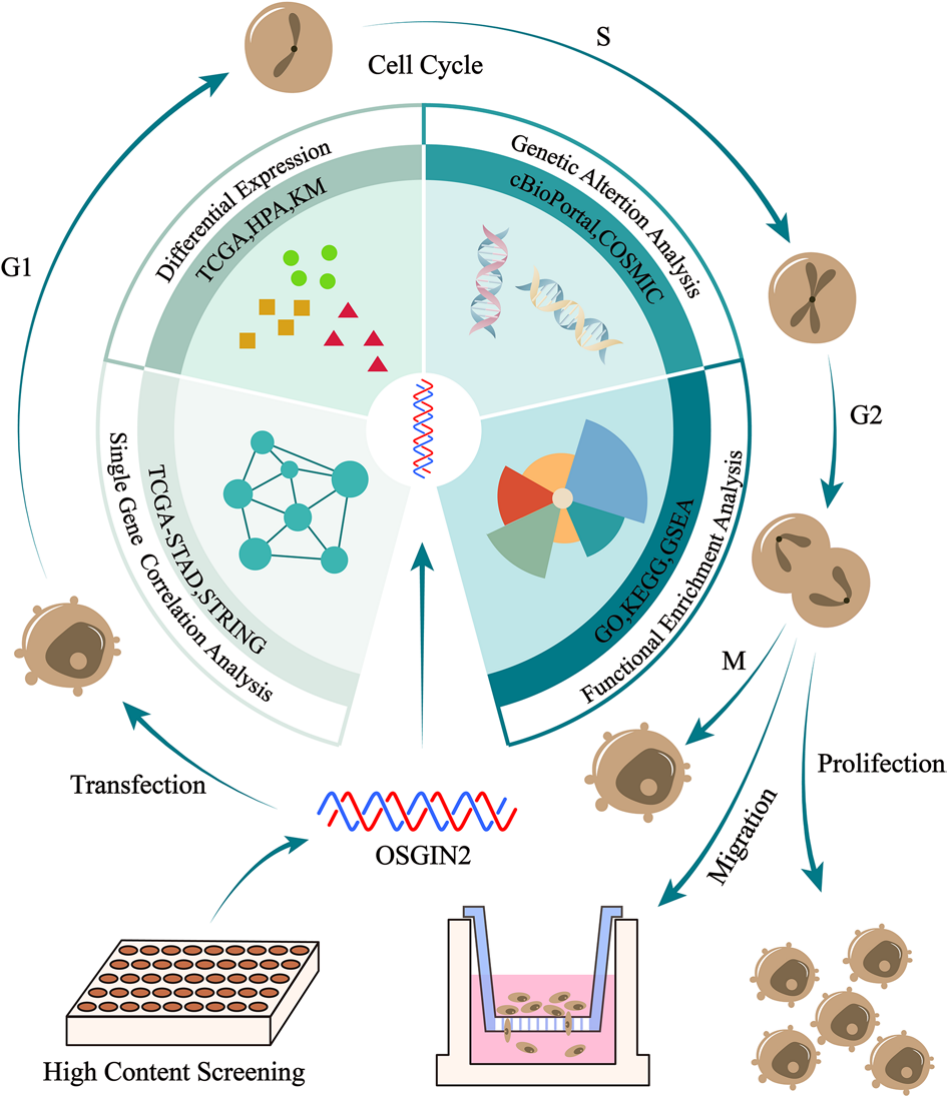
Clinical prognostic value of OSGIN2 in gastric cancer and its proliferative effect in vitro.

## Results

### OSGIN2 is a highly expressed differentially gene in GC and is associated with poor prognosis

**Figure 2:**
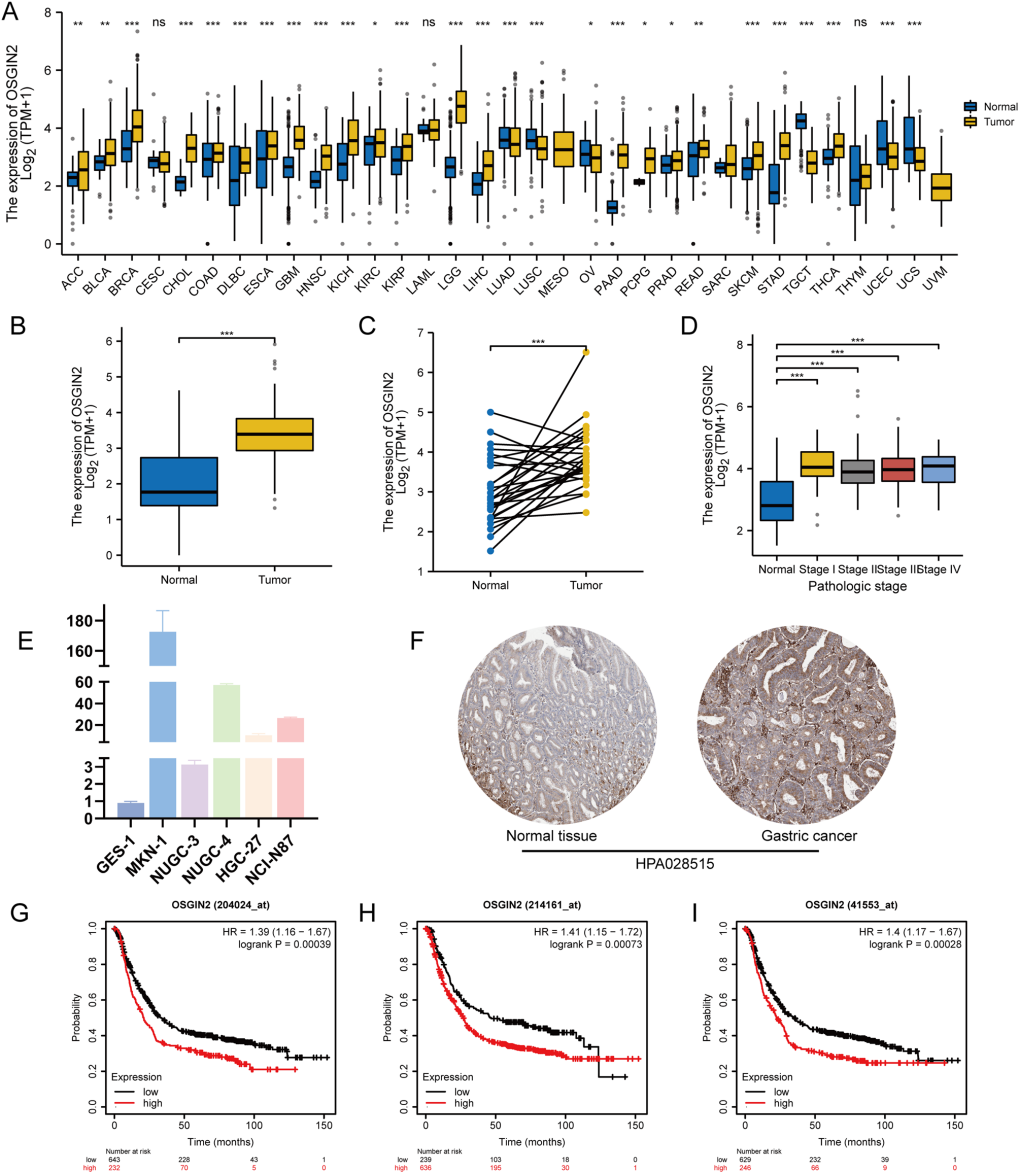
OSGIN2 is highly expressed in gastric cancer and indicates poor prognosis (**A**) Differential expression of OSGIN2 in TCGA pan-cancer. The results show increased expression of OSGIN2 in various tumor tissues. (**B**,**C**) Differences in the expression of OSGIN2 in TCGA-STAD. The results suggest higher expression of OSGIN2 in gastric cancer than in normal tissues. (**D**) The expression of OSGIN2 is higher in gastric cancer TNM stage I-IV than normal tissue. (**E**) RT-qPCR detection of differences in OSGIN2 expression in various cells. (**F**) The differences in the expression of OSGIN2 in different tissues were obtained from the tissue immunohistochemical results of the HPA public database, which shows high expression of OSGIN2 in gastric cancer tissues compared with normal tissues. (**G**–**I**) Prognostic analysis of OSGIN2 by KM database shows that high expression of OSGIN2 in three different gastric cancer microarray data indicates poor prognosis. ns *p* ≥ 0.05; * *p* < 0.05; ** *p* < 0.01; *** *p* < 0.001.

Through the comparing of OSGIN2 expression between tumors and the corresponding normal tissues in TCGA, the results demonstrated the increased expression level of OSGIN2 in various tumor tissues, including the gastric cancer group. Paired sample analysis of gastric cancer and normal tissues also showed high OSGIN2 expression in gastric cancer tissues (Fig. 2A–C). Although no significant differences of OSGIN2 expression were observed in different TNM stages of GC (Supplementary Table S1), the expression level of OSGIN2 in GC was still found to be significantly higher than that in normal tissues (Fig. 2D). In the comparison of OSGIN2 mRNA expression levels in 5 kinds of gastric cancer cells and 1 normal gastric epithelial cell, gastric cancer cells also displayed a significantly higher level (Fig. 2E). Immunohistochemical results from the HPA public database showed that OSGIN2 was higher expressed in gastric cancer tissues compared with normal tissues (Fig. 2F). In the three different gastric cancer microarray data from the KM database, the high expression of OSGIN2 decreased the survival time and affected the prognosis of GC patients (Fig. 2G–I).

### OSGIN2-related genes and interacting proteins

**Figure 3:**
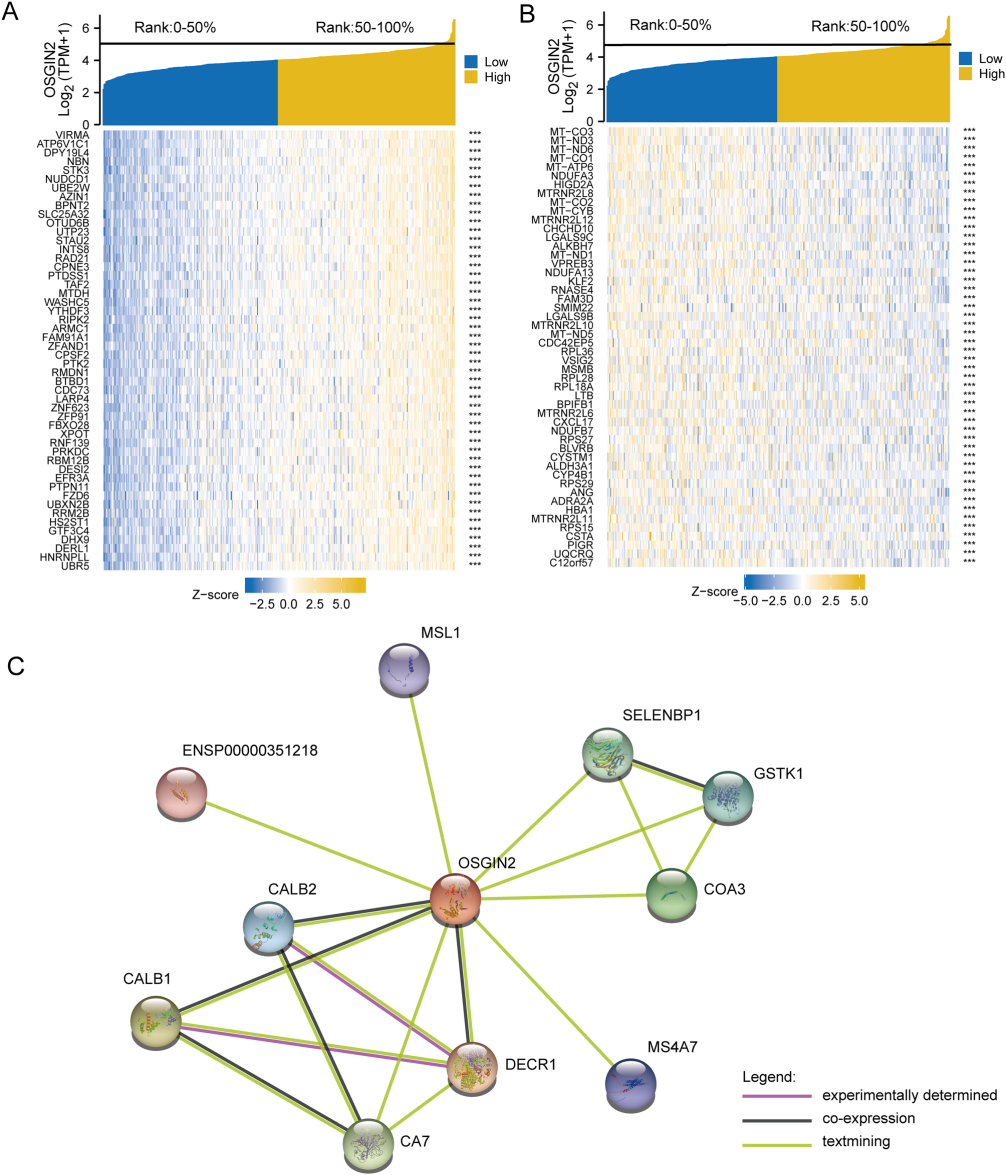
Single Gene Expression Correlation Analysis (**A**) The top 50 coding genes that are positively correlated with the expression of OSGIN2 at the mRNA level are obtained from STAD data in the TCGA database. (**B**) The top 50 coding genes that are negatively correlated with the expression of OSGIN2 at the mRNA level are obtained from STAD data in the TCGA database. (**C**) Using the STRING database to predict the protein–protein interaction network (PPI network) of OSGIN2. ns *p* ≥ 0.05; * *p* < 0.05; ** *p* < 0.01; *** *p* < 0.001.

To identify genes and proteins that are potentially associated with OSGIN2, we extracted co-expressed genes from the TCGA-STAD data and constructed a co-expression heat map based on the positive or negative correlation between genes and OSGIN2. Figure 3A,B showed the top 50 genes positively or negatively correlated with the mRNA expression levels of OSGIN2, respectively. The potential protein-coding genes related to OSGIN2 were retrieved through the STRING database. It was found that OSGIN2 had a protein interaction network relationship with MSL1, SELENBP1, GSTK1, COA3, MS4A7, DECR1, CA7, CALB1, CALB2, ENSP00000251218 (Fig. 3C).

### Potential functional enrichment analysis of OSGIN2

**Figure 4:**
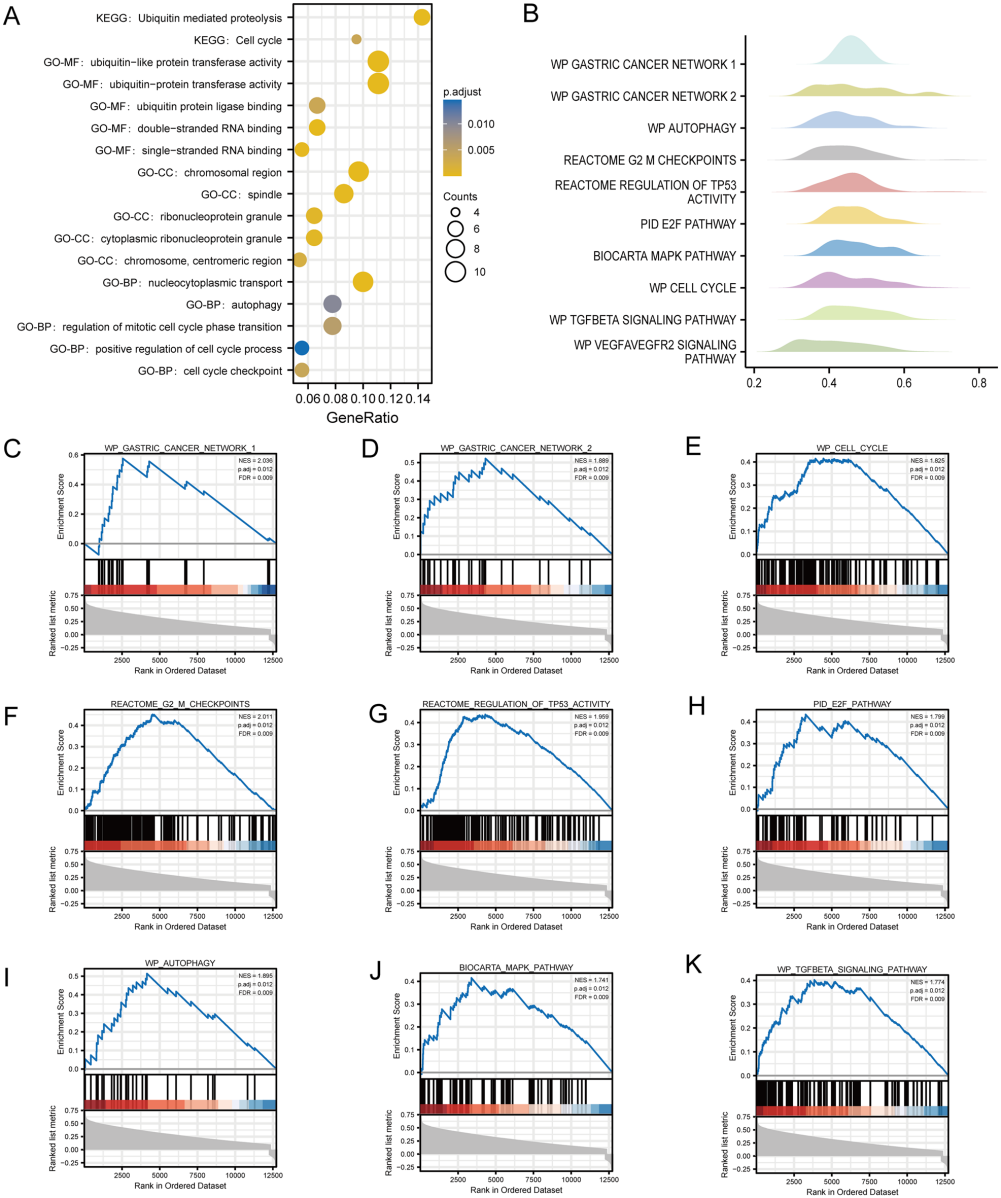
Functional enrichment analysis of OSGIN2-related genes (**A**) GO + KEGG^70–72^ functional enrichment analysis of DEGs and visualization of bubble diagram. (**B**) GSEA enrichment analysis was performed on DEGs, and 10 datasets related to gastric cancer were selected to depict mountain maps for visualization. (**C–K**) Dataset-specific enrichment score results related to gastric cancer in GSEA enrichment analysis results.

We included the genes co-expressed with OSGIN2 in the functional enrichment analysis of GO and KEGG, and the results revealed that a total of 231 BP, 30 CC, 39 MF, and 2 KEGG were co-enriched when the enrichment conditions were met (*p*. adj < 0.05 and Q value < 0.2). The bubble diagram showed some of the results (Fig. 4A). The biological functions of OSGIN2 may be mainly in ubiquitin-mediated proteolysis and cell cycle. The statistically significant (*p* < 0.05) coding genes in the correlation analysis with OSGIN2 were included in the Gene Set Enrichment Analysis (GSEA). The significant enrichment conditions were |NES|> 1, FDR (Q value) < 0.25, and *p*. adjust < 0.05. 317 data sets were obtained, of which 10 data sets related to GC were selected to depict the mountain map, including autophagy, G2_M checkpoint, regulation of TP53 activity, E2F pathway, MAPK pathway, cell cycle, TGFβ signaling pathway, etc. (Fig. 4B). They were 2 datasets directly related to gastric cancer, and the specific enrichment score was shown in Fig. 4C–K.

### OSGIN2 interferes with immunotherapy response of gastric cancer

**Figure 5:**
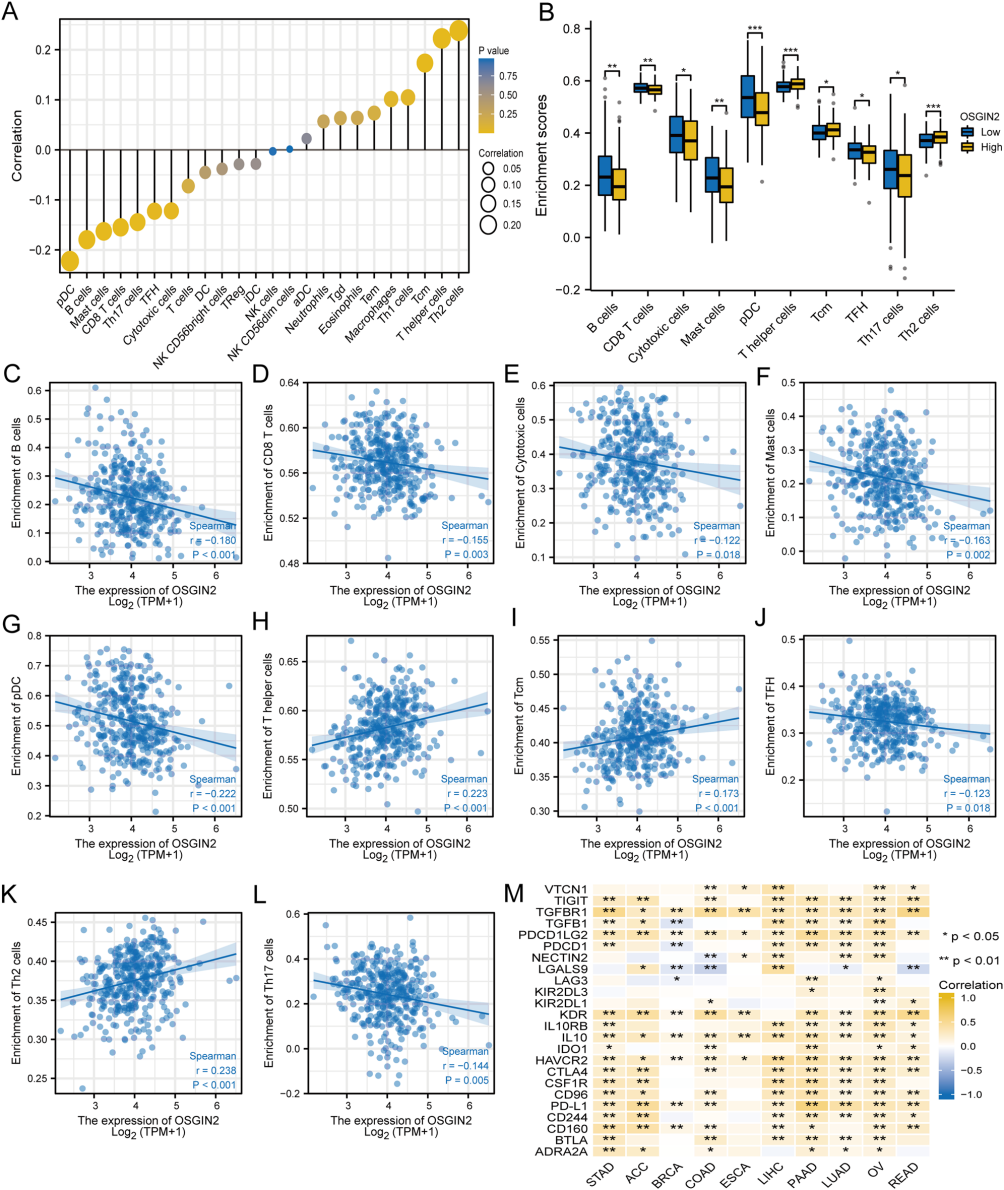
Correlation of OSGIN2 with immune infiltration (**A**) Lollipop diagram of the correlation between OSGIN2 and 24 types of immune cells. (**B**) The difference in the Infiltration fraction of 10 immune cells (Th2 cells, T helper cells, TCM, pDC, B cells, Mast cells, CD8 T cells, Th17 cells, TFH, and Cytotoxic cells) in the OSGIN2 high or low expression groups. (**C–L**) Correlation coefficient between the infiltration levels of the above 10 immune cells and OSGIN2 expression in gastric cancer. (M) Immune-related biomarkers correlate with OSGIN2 expression in multiple tumors. ns *p* ≥ 0.05; * *p* < 0.05; ** *p* < 0.01; *** p < 0.001.

Figure 5A was the lollipop diagram of OSGIN2 and tumor-associated immune cell infiltration, displaying the connection between OSGIN2 and 24 kinds of immune cells in various cancers. According to the results, OSGIN2 has a significantly positive correlation with the infiltration of Th2 cells, T helper cells, and TCM immune cells, and a significantly negative correlation with pDC, B cells, mast cells, CD8 T cells, Th17 cells, TFH, and cytotoxic cells. The differences in the infiltration fraction of these 10 kinds of immune cells between the high and low OSGIN2 expression groups were shown in Fig. 5B. Spearman method was also used to list the correlation coefficients and *p*-value between the infiltration levels of the above 10 immune cells and OSGIN2 expression in GC, and significant associations were observed (Fig. 5C–L). Pan-cancer analysis revealed that most of the current clinical immune-related biomarkers, including PD-L1 and CTLA4, were associated with OSGIN2 (Fig. 5M).

### Mutation of OSGIN2 in gastric cancer

**Figure 6:**
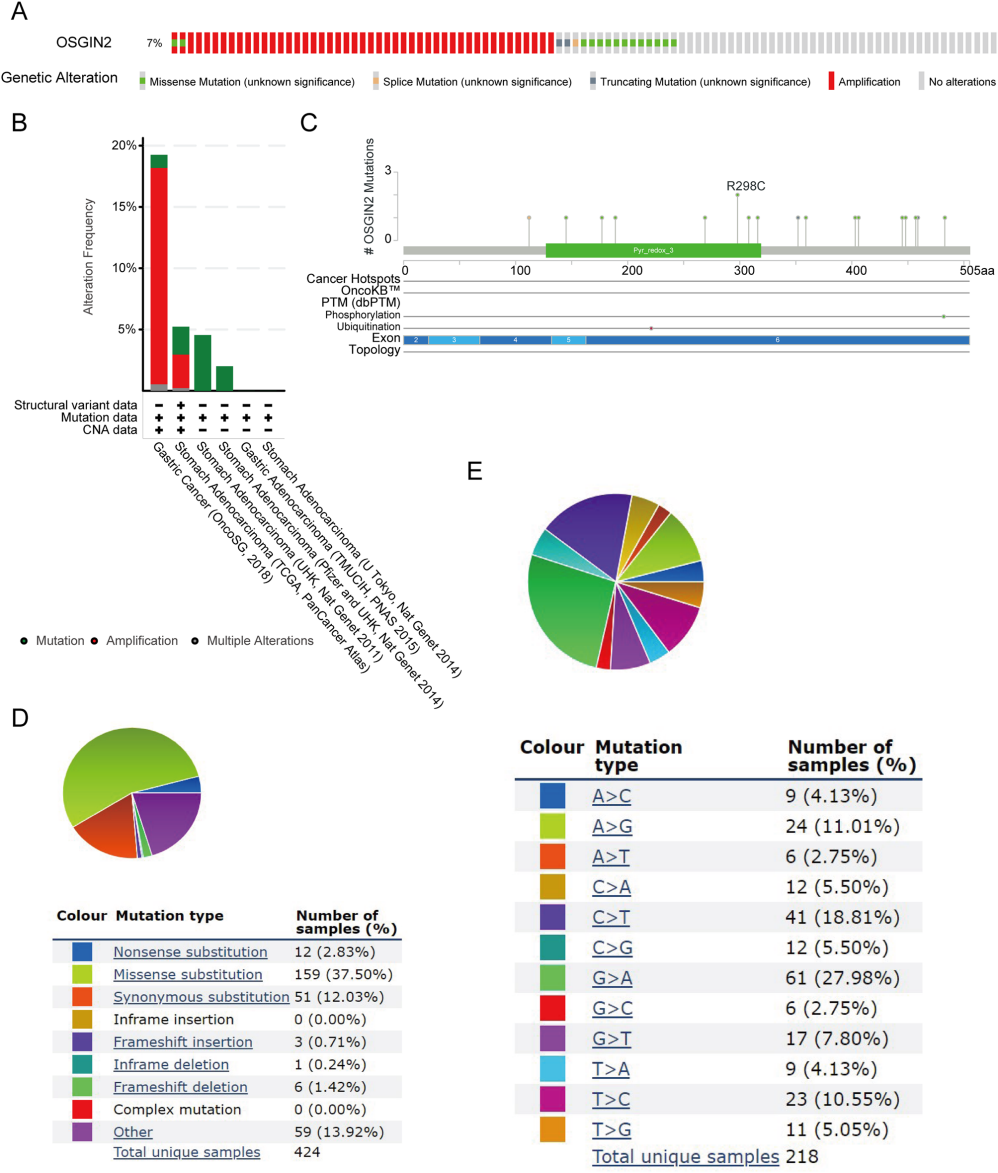
Mutation information of OSGIN2 in gastric cancer (**A**,**B**) Mutation frequency and mutation type of OSGIN2 in gastric cancer, assessed using the cBioPortal database. (**C**) Specific sites where OSGIN2 is mutated and specific sites of phosphorylation and ubiquitination in GC patients. (D,E) Mutation types of OSGIN2 assessed in the COSMIC database.

The cBioPortal database was utilized to evaluate the mutation frequency of OSGIN2 in gastric cancer. Six datasets (TMUCIH, OncoSG, Pfizer and UHK, U Tokyo, UHK, and TCGA-PanCancer Atlas), including 857 samples, were used for this analysis. A total of 7% (62/857) of GC patients had genetic alterations in OSGIN2, with the most common type of alteration being amplification, followed by mutation. The most common type of mutation was missense mutation, which was only 1.6% (14/857) due to the low mutation frequency (Fig. 6A,B). We didn’t observe a significant association between OSGIN2 mutation and the prognosis of GC patients. Figure 6C shows the specific mutation sites, including phosphorylation and ubiquitination sites, of OSGIN2 in GC patients. Further investigation of OSGIN2 mutation types in the COSMIC database revealed that about 37.5% of the samples had missense substitutions, 12.03% of the samples had synonymous substitutions, and 2.83% had nonsense substitutions. (Fig. 6D,E). Observed substitution mainly occurred in G > A (27.98%), C > T (18.81%), A > G (11.01%) and T > C (10.55%).

### Functional verification in vitro

**Figure 7:**
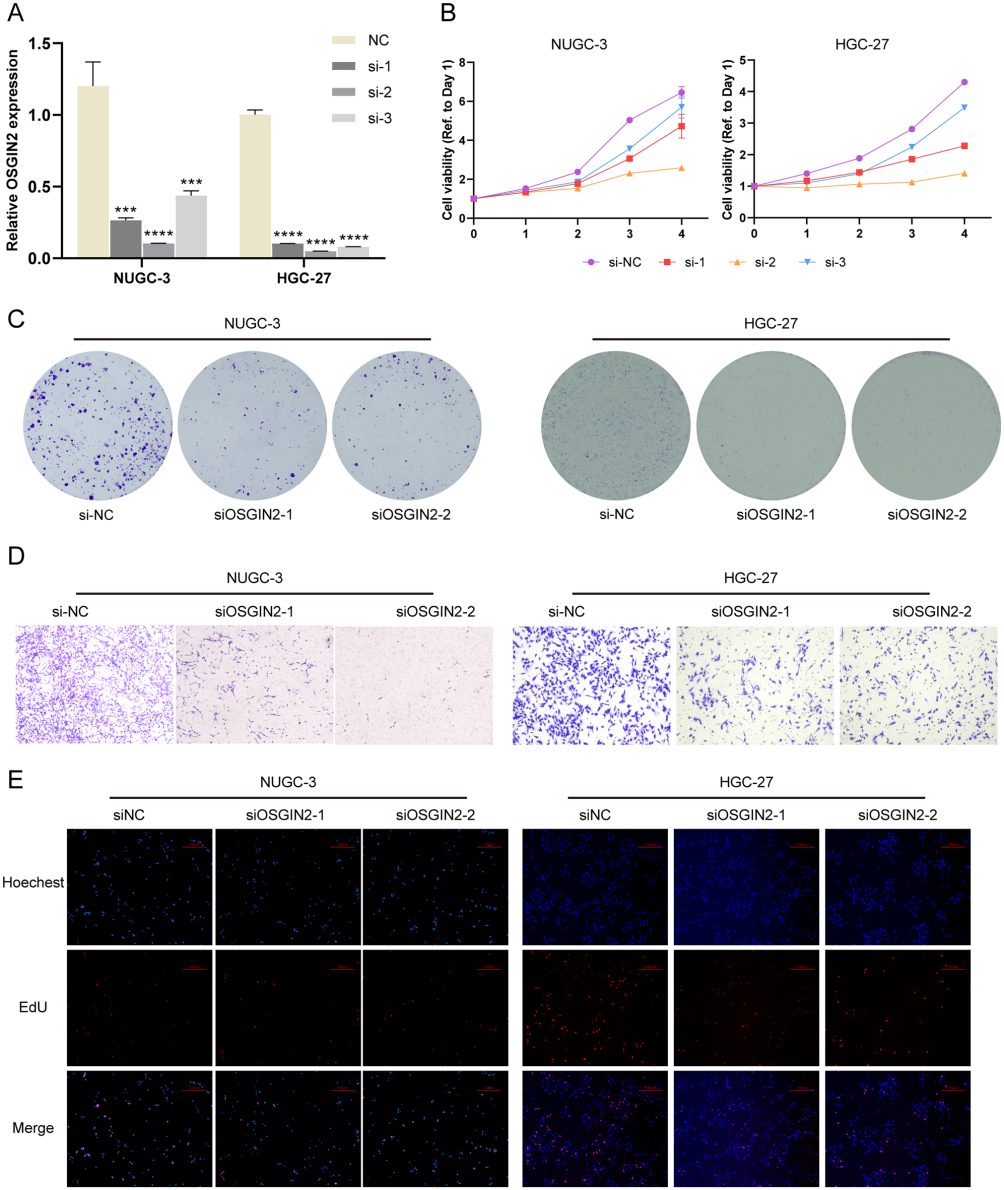
Functional verification of proliferation and migration of OSGIN2-knockdown NUGC3 and HGC27 cells (**A**) Transfection efficiency of OSGIN2 siRNA. (n = 3) (**B**) Cell proliferation ability of OSGIN2-knockdown cells, analyzed by CCK8 assays. (n = 3) (**C**) Colony formation ability of OSGIN2-knockdown cells. (n = 3) (**D**) Migration capacity of OSGIN2-knockdown cells. (n = 3) (**E**) Cellular DNA replication activity of OSGIN2-knockdown cells, detected by EdU experiment. (n = 3) ns* p* ≥ 0.05, * *p* < 0.05, ** *p* < 0.01, *** *p* < 0.001, **** *p* < 0.0001 vs si-NC group.

**Figure 8:**
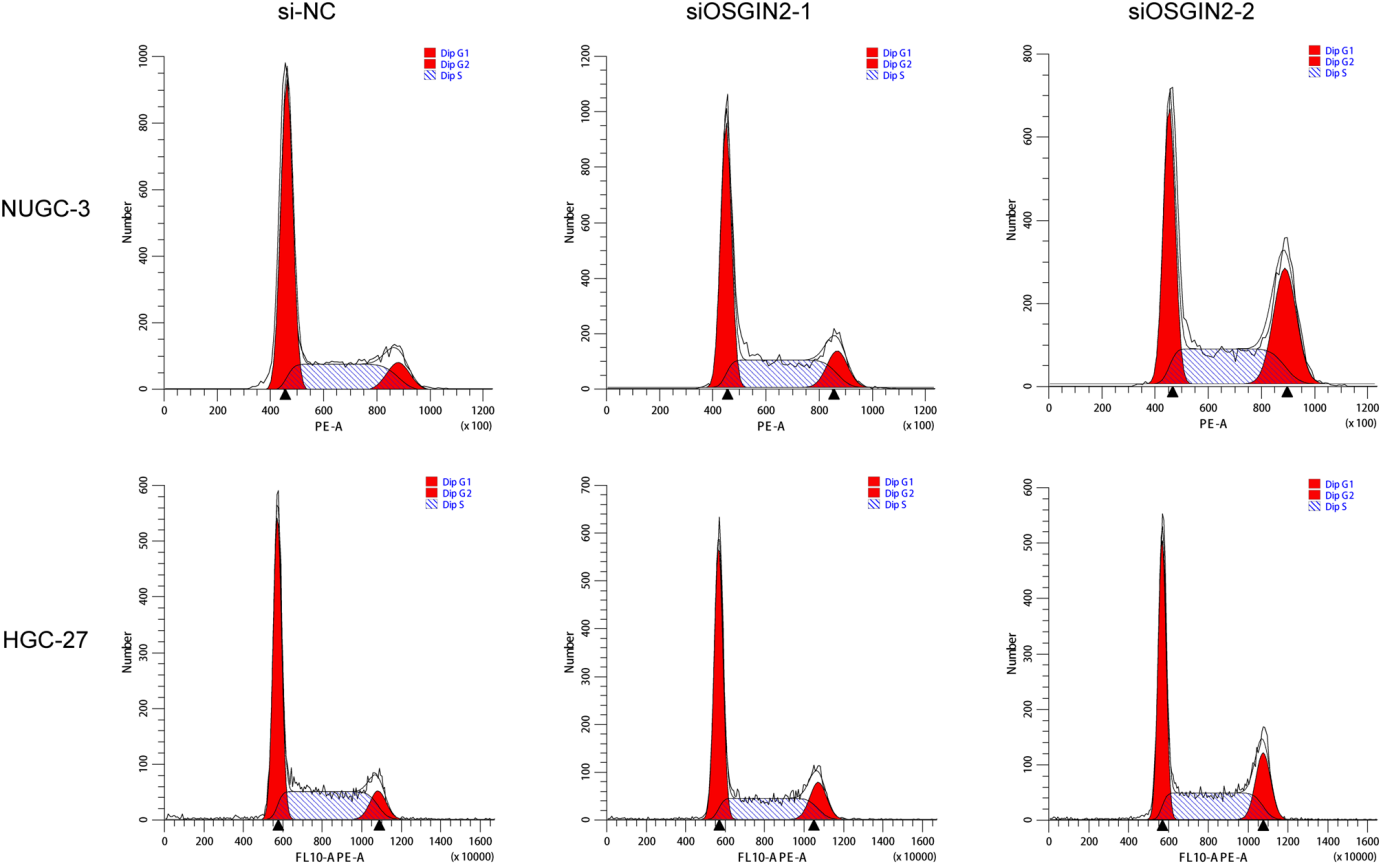
The role of OSGIN2 on cell cycle regulation Cell cycle of OSGIN2-knockdown NUGC3 and HGC27 cells. (n = 3).

Finally, we conducted functional experiments to validate the promoting effect of OSGIN2 on gastric cancer cells. After transfecting siRNAs and verifying their transfection efficiency (Fig. 7A), we determined that the knockdown of OSGIN2 inhibited NUGC3 and HGC27 cells proliferation, as shown by CCK8 assay (Fig. 7B). Based on the transfection efficiency, siOSGIN2-1 and siOSGIN2-2 were selected for subsequent experiments. Figure 7C showed that the colony formation ability of the OSGIN2 knockdown cells was reduced, and EdU experiments further confirmed the effect of OSGIN2 on cellular DNA replication activity (Fig. 7E). We also demonstrated that the knockdown of OSGIN2 may reduce cell migration capacity (Fig. 7D). In addition, we explored the role of OSGIN2 in cell cycle regulation, and the results suggested that OSGIN2 knockdown would cause DNA damage in tumor cells and an increase in cells stuck in the G2/M phase (Fig. 8).

## Discussion

Gastric cancer is a cancer with high-risk factor and is highly prevalent worldwide^1^. Despite greatly improvements in diagnostic and therapeutic tools over the past fifty years, GC remains highly malignant and there are still problems such as poor survival prognosis^19,20^. Therefore, it is crucial to investigate the pathogenesis of GC and identify markers for its diagnosis and prognosis. OSGIN2 is located on chromosome 8q21.3 with gene synonym C8orf1/HT41^11^ and the gene transcription product is a 56.7 kDa protein containing 505 amino acids^12^. Researches have shown that OSGIN2 may be involved in the physiological processes of cancer and other diseases^13,14^. Chen Z et al.^21^ also observed high expression of OSGIN2 in colorectal cancer tissues. Our study confirmed the association of OSGIN2 with gastric cancer and suggested it as a potential marker for the diagnosis and prognosis of GC by bioinformatics analysis and experimental validation.

In our research, the upregulated of OSGIN2 was found in various cancers, including gastric cancer, glioblastoma, and pancreatic cancer. Moreover, the OSGIN2 expression level in gastric cancer was increased in all stages I-IV compared with normal tissues. The higher expression of OSGIN2 was also observed at the cellular level. Hence, we suggested that GC might be connected with the upregulation of OSGIN2. In addition, the high expression of OSGIN2 in different gastric cancer microarray data suggested a poor prognosis. Therefore, we thought OSGIN2 might be an important biomarker for gastric cancer diagnosis, prognosis, and even one of the key factors involved in tumor progression.

In the single gene expression correlation analysis, we discovered several significant positive associations between OSGIN2 and certain genes, including AtP6V1C1, STK3, RIPK2, and PTK2 at the mRNA expression level. AtP6V1C1 has been shown to promote breast cancer cell growth by upregulating V-ATPase activity and activating the mTORC1 pathway^22^. STK3, by regulating the cell cycle, could accelerate the progression of gastric cancer and serve as a prognostic biomarker in GC^23^. RIPK2 contributes to both proliferation and invasion in cancers such as ovarian and gastric cancers, mediates NOD1 to regulate the NF-κB pathway, and promotes immunotherapy resistance by triggering cytotoxic T lymphocyte dysfunction, which is highly detrimental to prognosis^24,25^. Cyclic RNA PTK2 can accelerate the proliferation of gastric cancer cell and inhibit apoptosis via miR-139-3p^26,27^. Meanwhile, we found LGALS9C and FAM3D were negatively associated with OSGIN2. LGALS9C is a proven tumor suppressor gene^28^ and exhibits a negative correlation with immune infiltrates^29^. FAM3D could inhibit colon cancer development by NF-κB signaling pathway^30^. Additionally, a number of studies have shown that CALB2 promotes the generation and development of various cancer cells, including colon cancer and pancreatic cancer, and is closely related to cancer cell migration^31–33^.

Research has demonstrated that OSGIN2 is associated with oxidative stress, serving as a key target gene of specific microRNA in cellular stress response^34^. SATTA S et al. identified that overexpression of OSGIN2 leads to cell cycle arrest and the induction of senescence^35^. Among the PPI interaction network of OSGIN2, proteins such as MSL1, CALB2, SELENBP1, GSTK1, COA3, MS4A7, and DECR1 were closely related to OSGIN2. MSL1, having a DNA-repairing activity, can suppress DNA damage-induced apoptosis to promote cancer cell survival^36^. WEI T et al.^37^ found that MSL1 is involved in cell proliferation and the EMT process, and its malfunctions will cause changes in the cell cytoskeleton and morphology, promoting the EMT process and metastasis. While SELENBP1 is a member of the selenium-binding protein family, known for its potent anti-cancer properties^38^. Research have revealed that SELENBP1 participates in the regulation of oxidative stress and the decreased expression of it could promote tumor growth^39^ and invasiveness^40^. GSTK1, which is mainly expressed in the mitochondria, can protect cells against exogenous and endogenous oxidative stress in the mitochondria^41^. COA3 plays an important role in negative feedback regulation of COX1 translation in mitochondria^42^, which is related to the migration and invasion of gastric cancer cells^43^. DECR1 inhibits ferroptosis in prostate cancer, which is driven by the reduction of scavenging and the iron-dependent accumulation of ROS^44^. In addition, the low expression of MS4A7 was found to be correlated with better overall survival (OS) in gastric cancer patients^45^. Our study demonstrated that OSGIN2 may be functionally linked to cancer formation and development, and suggested upstream and downstream genes that may be related. However, the specific relations and functional roles are still unknown. Therefore, further research should be conducted to explore the contribution of OSGIN2 and these proteins to GC.

Through GO and KEGG bio enrichment analysis, several pathways were found closely associated with OSGIN2, including KEGG: Cell cycle, GO-BP: Cell cycle checkpoint, GO-BP: Regulation of mitotic cell cycle phase transition, and other pathways related to the cell cycle. Aberrant genes expression in cancer cells is directly involved in regulating cell cycle, and cell cycle dysfunction results in excessive cell proliferation and low apoptosis rates, ultimately leading to oncogenesis. In the GSEA enrichment analysis, MAPK pathway, E2F pathway, cell cycle, and TGFBETA pathway were found to be related to gastric cancer^46–49^. MAPK pathway, a common transduction pathway, is involved in various aspects of cancer progression, including proliferation, apoptosis, and immune escape^50^. JIANG T et al.^51^ discovered that the inhibition of MAPK1 and its downstream factors could inhibit the proliferation and invasion of GC cells. E2F pathway is important in cell cycle regulation and takes part in angiogenesis, extracellular matrix remodeling, and tumor cell-endothelial cell interactions^52–55^. The TGFBETA signaling pathway consists of a series of pathways, which is mediated by transforming growth factor-mediated series of signaling processes. It is crucial for cell proliferation, apoptosis, mesenchymal production, inflammatory response, and immune function^56^, and its mis-regulation can lead to tumor development^57^. From the above, it can be speculated that OSGIN2 may promote the proliferation and invasion of gastric cancer cell by interfering with the cell cycle.

We also observed a link between OSGIN2 expression and immune cells infiltration. Tumor infiltration of immune cells is associated with tumor development, metastasis, etc.^58^. Our results showed that the expression of OSGIN2 was positively related with 3 types of immune cells, namely T helper cells, Th2 cells, and TCM, while negatively correlated with immune cells, such as CD8 T cells, B cells, TFH, and Cytotoxic cells. Immune cells may have a dual function in cancer. Research has found that T helper cells could produce a variety of factors that affect the tumor antigen-specific cytotoxic T cell (CTL) response and further promoted antitumor immunity^59^. Th2 cells have been reported to promote the immune escape of urological tumors^60^. CD8 T cells are considered the main anti-cancer cells, which produce CTL to kill specific pMHC complexes in cancer cells^61^. While B cells play a part in antigen presentation, immunological regulation, and the humoral immune response by generating cytokine^62^. TFH differentiation is associated with the differentiation and coordinated production of IL-21 and IL-4, and may also signal B-cell differentiation through regulating transcription factors such as Bcl-6 and Blimp-1^63–65^, while IL-21 is potentially beneficial in cancer immunotherapy strategies through CD8 T-cell responses. Thus, high expression of OSGIN2 in gastric cancer may affect tumor immunity and lead to carcinogenesis.

To address the correlation between OSGIN2 mutation and cancer progression, we evaluated the frequency of mutations in OSGIN2 in gastric cancer. Results showed that amplification and mutation occurred predominantly in GC. Amplification implies that OSGIN2 has a higher expression, i.e., the increased expression of OSGIN2 in GC may be partly contributed by DNA copy number amplification. The results from COSMIC showed that missense substitution occurred in about 37.5% of the samples, which may act synergistically with cancer by affecting protein stability, conformation, interactions, and catalytic activity^66^.

Furthermore, the cell experiments demonstrated that the knockdown of OSGIN2 in NUGC3 and HGC27 cells inhibited cell proliferation, migration, and DNA replication, caused DNA damage and an increase of cells stuck in the G2/M phase.

In conclusion, here we are the first to indicate the diagnostic and prognostic value of OSING2 in GC, providing a new research direction for the molecular mechanism of gastric carcinogenesis and development, as well as a new idea for the treatment and prognosis of gastric cancer. Although we did cellular experiments to validate the findings of our series of bioinformatics data analyses from online databases, our study still has some limitations. Firstly, to obtain more accurate results, it is still necessary to further expand the size of sample and improve the quality of the data used. Secondly, a large number of clinical samples are needed to carry on the comprehensive verification. Therefore, further experiments in vitro/in vivo, clinical cohort studies, and more in-depth mechanistic studies are needed to validate our findings.

## Conclusions

In summary, this research identifies OSGIN2 as a possible gene associated with gastric cancer progression through a combination of bioinformatics analysis and cellular experiments, and expects it to be a promising therapeutic target for improving the therapeutic efficacy and prognosis of gastric cancer. OSGIN2 was significantly highly expressed in gastric cancer and was able to serve as a predictor of prognosis. Functionally, OSIGIN2 may be associated with cell cycle and autophagy, and involved in regulating signaling pathways such as E2F pathway and MAPK pathway. The knockdown of OSGIN2 significantly inhibited cell proliferation and migration in vitro.

## Methods

### Data source and preprocessing

To assess OSGIN2 expression in the pan-cancer and stomach adenocarcinoma (STAD), data and selected samples in tumor tissues (RNASeq-TPM) were downloaded from the TCGA database (https://portal.gdc.cancer.gov). A combination analysis of the TCGA and Genotype-Tissue Expression (GTEx) databases (https://gtexportal.org/) were performed on the normal tissue samples. The characteristics of GC patients in the TCGA database are summarized in Supplementary Table S1.

### OSGIN2 differential expression in GC

Differential expression of OSGIN2 in mRNA level was carried out by R software (3.6.3 version) and the results were shown by a box diagram and paired sample wiring plots. OSGIN2-high or OSGIN2-low stranded for the statistical rankings for the expression of OSGIN2 above or below the median value, respectively.

The differential expression of OSGIN2 in protein level was explored through the Human Protein Atlas (HPA) database (http://www.proteinatlas.org/)^67^, and immunohistochemically stained by HPA028515 antibody.

### Kaplan–Meier plot analysis

The Kaplan–Meier plot (http://kmplot.com/analysis/) was used to analyze the relationship between the expression of the OSGIN2 gene and survival rates in three separate gastric cancer microarray data sets (204,024, 214,161, and 41,553) based on hazard ratios (HR) and log-rank *p*-values.

### Correlation and enrichment analyses

Using TCGA-STAD data, a Spearman correlation study of OSGIN2 mRNA and other mRNAs in gastric cancer was carried out. The heat map analysis was performed on 50 genes, which are the most positively or negatively linked to OSGIN2. These 100 genes were also selected for Gene ontology (GO) analysis^68^ and Kyoto Encyclopedia of Genes and Genomes (KEGG) analysis^69–72^ using the EnrichGO and EnrichKEGG functions from the clusterProfiler [3.14.3] package^73^ in R software to determine the function of OSGIN2. Statistical significance was defined as *p*.adj < 0.05 and Q value < 0.2. The GO terms were divided into three categories, namely biological processes (BP), cellular composition (CC), and molecular function (MF).

Furthermore, coding genes that had statistical significance in the OSGIN2 expression spearman correlation analysis were selected for Gene Set Enrichment Analysis (GSEA)^74^. To predict phenotypes and signal pathways related to OSGIN2, GSEA began with the OSGIN2 differentially expressed matrix and analyzed the differences in signal pathways between the OSGIN2-high and OSGIN2-low groups. Reference gene set was the functional set c2.cp.v7.2.symbols.gmt [Curated] from MSigDB Collections gene set database. Enrichment is defined as significant when *p* < 0.05, False discovery rate (FDR) < 0.25, and normalized enrichment score (|NES|) > 1.

### Protein–protein interaction (PPI) network analysis

The STRING database (http://stringdb.org) was used to build the protein–protein interaction (PPI) network of OSGIN2.^75^. PPI pairs with interaction scores > 0.40 were used to construct PPI networks.

### Immune cell infiltration analysis

Single-sample GSEA (ssGSEA) method in the R package GSVA (version 3.6)^76^ was utilized to investigate the molecular characterization under tumor immune interactions in GC. Gene expression profiling data from the literature was used to examine the effect of OSGIN2 expression on immune cell infiltration, and the *p*-values were determined using Wilcoxon rank-sum and Spearman's rank correlation tests. For statistically significant immune cells, the correlation with OSGIN2 expression was visualized using a grouped boxplot. And the specific correlation of each cell with OSGIN2 expression was listed by scatter plots. In addition, Spearman correlation analysis with OSGIN2 was performed for some immune-related biomarkers in various tumor types, and these biomarkers were listed in the heat map matrix.

### Genetic alteration analysis of OSGIN2

The genetic alteration frequency, mutation type, mutation sites, and gene expression modification sites of OSGIN2 in gastric cancer were evaluated by cBioPortal (http://www.cbioportal.org/)^77^. The OSGIN2 mutation types of GC were further assessed by the Catalogue of Somatic Mutations in Cancer (COSMIC) database (http://cancer.sanger.ac.uk^78^.

### Cell culture

Gastric cancer cells NUGC3 and HGC27 were obtained from the National Collection of Authenticated Cell Cultures and cultured in RPMI-1640 medium (Biological Industries, Israel), which was supplemented with 10% fetal bovine serum (FBS, MEILUNCELL, China), 100 mg/mL penicillin, and 100 mg/mL streptomycin (Biological Industries, Israel). Cells were cultured in an incubator with 5% CO_2_ at 37 °C. Depending on the status of the cells' development, the medium was periodically adjusted. Cells in the logarithmic growth phase were used for subsequent experiments.

### siRNA interference

Logarithmic-phase cells were seeded into 6-well plates. Target siRNA was transfected by jetPRIME kit (Polyplus Transfection) after adhering overnight. The normal medium was replaced after 24 h, and digestion was performed after 48 h to collect cells for subsequent experiments. The siRNA sequences used were as follows: hOSGIN2 si-1 sense 5'-3' (GCUCGCUACUAUAAACAUUAUUTT) and antisense 5'-3' (AUAAUGUUUAUAGUAGCGAGCTT), hOSGIN2 si-2 sense 5'-3' (GCAGACGAGUAACUGAUCCAATT) and antisense 5'-3' (UUGGAUCAGUUACUCGUCUGCTT), hOSGIN2 si-3 sense 5′-3′ (CCUGCCCAUCUGGAAAUUGAATT) and antisense 5′-3′ (UUCAAUUUCCAGAUGGGCAGGTT).

### Plate colony formation experiment

After treatment of cells in 6-well plates, the seeded cells were digested at 1000 cells/well, the medium was changed every 3 days and fixed with 4% paraformaldehyde (Beyotime, China) after 2 weeks. The number of clones was observed after crystal violet staining.

### CCK8 experiment

Logarithmic-phase cells were inoculated into 96-well plates with 3000 cells/well and cultured in the incubator with 5% CO_2_ at 37 °C. At a ratio of 1:9, 10 µL CCK-8 solution (MedChemExpress, USA) was added to each well. After incubation in the incubator for 1 h, a microplate reader was used to measure the absorbance (d0-d4) of each well at 450 nm.

### Detection of EdU proliferation level

After cells were seeded in confocal dishes with 24 h of adherent culture, the EdU kit (Beyotime, China) was utilized to measure cells proliferation under the manufacturer's instructions. After fixing, apollo staining, and hochest staining the cells, the number of EdU positive cells was observed by fluorescence microscope.

### Cell transwell experiment

After being digested and washed, the cells were adjusted to 6 × 10^5^ cells/ml, 200 μl of cell suspension was added to the upper chamber, while 500 μl of medium with 10% FBS were added to the lower chamber, respectively. After being placed in the incubator at 37 °C with 5% CO2 for 48 h, the chamber was moved out, fixed with 4% paraformaldehyde, stained with crystal violet, and finally photographed under a microscope.

### RNA extraction and real-time quantitative PCR

FastPure Cell/Tissue Total RNA Isolation Kit V2 (Vazyme Biotech, China) was used to extract the total cellular RNA. 1 μg of RNA was taken for reverse transcription by HiScript II Q RT SuperMix for qPCR (Vazyme Biotech, China). ChamQ Universal SYBR qPCR Master Mix (Vazyme Biotech, China) was utilized to detect the RNA expression level of OSGIN2, with β-actin was used as an internal reference. The primer sequences used were as follows: β-actin, 5′-CATCCACGAAACTACCTTCAACTCC-3′(Forward) and 5′-GAGCCGCCGATCCACACG-3′(Reverse); OSGIN2, 5′-TGTTGACAATCAGCTTTGGAAGT-3′ (Forward) and 5′-CCTTTTAGGCTCCTTCGTTTACT-3′(Reverse).

### Statistical analysis

All bioinformatics analyses in this study were conducted using R software (Version 3.6.3, https://cran.r-project.org/src/base/R-3/R-3.6.3.tar.gz) and multiple data packets, including clusterProfiler (version 3.14.3) and GSVA (version 3.6). The means ± standard deviation from three separate experiments was used to represent all experimental data. GraphPad Prism (version 8.4) and SPSS (version 22.0) were used in statistical analysis. One-way ANOVA or Student's t-test was used for the comparison of different groups. *p*-value < 0.05 was considered statistically significant.

## Supplementary Information

Supplementary Information.

## Data Availability

The datasets analysed during the current study are available in the TCGA database and Genotype-Tissue Expression (GTEx) databases (https://toil-xena-hub.s3.us-east-1.amazonaws.com/download/TcgaTargetGtex_rsem_gene_tpm.gz), Human Protein Atlas database (https://www.proteinatlas.org/ENSG00000164823-OSGIN2), Kaplan–Meier plot (204,024, 214,161, and 41,553), STRING database (https://cn.string-db.org/cgi/network?taskId=bhCXD2HUDhct&sessionId=by3fs8PAf0JU), Catalogue of Somatic Mutations in Cancer (COSMIC) database (OSGIN2_ENST00000297438), and cBioPortal database (URL https://bit.ly/3Ih2OoX).
