# Supplementary material for: Clinical prognostic value of OSGIN2 in gastric cancer and its proliferative effect in vitro
Source: Sci Rep. 2023 Apr 8;13:5775. doi: 10.1038/s41598-023-32934-5 (PMC10082810; doi:10.1038/s41598-023-32934-5)
Supplement: Supplementary file 1 — Supplementary Information. [file 41598_2023_32934_MOESM1_ESM.docx]

**Supplementary Table S1: The characteristics of gastric cancer patient in TCGA**

| Characters | level | Overall |
| --- | --- | --- |
| n |  | 375 |
| T stage (%) | T1 | 19(5.2%) |
|  | T2 | 80(21.8%) |
|  | T3 | 168(45.8%) |
|  | T4 | 100(27.2%) |
| N stage (%) | N0 | 111(31.1%) |
|  | N1 | 97(27.2%) |
|  | N2 | 75(21.0%) |
|  | N3 | 74(20.7%) |
| M stage (%) | M0 | 330(93.0%) |
|  | M1 | 25(7.0%) |
| Pathologic stage (%) | Stage I | 53(15.1%) |
|  | Stage II | 111(31.5%) |
|  | Stage III | 150(42.6%) |
|  | Stage IV | 38(10.8%) |
| Tumor status (%) | Tumor free | 214(63.5%) |
|  | With tumor | 123(36.5%) |
| Primary therapy outcome (%) | CR | 231(72.9%) |
|  | PD | 65(20.5%) |
|  | PR | 4(1.3%) |
|  | SD | 17(5.4%) |
| Gender (%) | Female | 134(35.7%) |
|  | Male | 241(64.3%) |
| Race (%) | Asian | 74(22.9%) |
|  | Black or African American | 11(3.4%) |
|  | White | 238(73.7%) |
| Age (%) | <=65 | 164(44.2%) |
|  | >65 | 207(55.8%) |
| Histological type (%) | Diffuse Type | 63(16.8%) |
|  | Mucinous Type | 19(5.1%) |
|  | Not Otherwise Specified | 207(55.3%) |
|  | Papillary Type | 5(1.3%) |
|  | Signet Ring Type | 11(2.9%) |
|  | Tubular Type | 69(18.4%) |
| Residual tumor (%) | R0 | 298(90.6%) |
|  | R1 | 15(4.6%) |
|  | R2 | 16(4.9%) |
| Histologic grade (%) | G1 | 10(2.7%) |
|  | G2 | 137(37.4%) |
|  | G3 | 219(59.8%) |
| Anatomic neoplasm subdivision (%) | Antrum/Distal | 138(38.2%) |
|  | Cardia/Proximal | 48(13.3%) |
|  | Fundus/Body | 130(36.0%) |
|  | Gastroesophageal Junction | 41(11.4%) |
|  | Other | 4(1.1%) |
| Reflux history (%) | No | 175(81.8%) |
|  | Yes | 39(18.2%) |
| Anti-reflux treatment (%) | No | 142(79.3%) |
|  | Yes | 37(20.7%) |
| Barrett’s esophagus (%) | No | 193(92.8%) |
|  | Yes | 15(7.2%) |
| TP53 status (%) | Mut | 172(46.2%) |
|  | WT | 200(53.8%) |
| PIK3CA status (%) | Mut | 59(15.9%) |
|  | WT | 313(84.1%) |
| Age (median [IQR]) |  | 67.00[58.00,73.00] |
